# Comparing Treatment Outcomes of Eligible Patients Consenting to or Declining Randomization in a Randomized Clinical Trial: A Secondary Analysis of the FISH Trial on Humeral Shaft Fractures

**DOI:** 10.2106/JBJS.OA.24.00018

**Published:** 2024-12-02

**Authors:** Thomas Ibounig, Cyrill Suter, Bakir O. Sumrein, Antti P. Launonen, Tomasz Czuba, Teppo L.N. Järvinen, Simo Taimela, Mika Paavola, Lasse Rämö, and the FISH Investigators

**Affiliations:** 1 Finnish Centre for Evidence-Based Orthopaedics (FICEBO), University of Helsinki, Helsinki, Finland; 2 Department of Orthopedics and Traumatology, Helsinki University Hospital, University of Helsinki, Helsinki, Finland; 3 Department of Orthopedics and Traumatology, Tampere University Hospital, University of Tampere, Tampere, Finland; 4 Lund University, Lund, Sweden

## Abstract

**Background:**

The Finnish Shaft of the Humerus (FISH) trial compared open reduction and internal plate fixation (ORIF) with functional bracing in adult patients with displaced, closed humeral shaft fractures. Here, we compare the results of the patients in the randomized clinical trial (RCT [the randomized cohort]) with those of the cohort of patients who were also eligible but declined randomization (the nonrandomized cohort) to investigate if patients’ treatment preference was associated with the outcomes during a 2-year follow-up.

**Methods:**

A total of 321 patients were treated at 2 university hospitals in Finland between November 2012 and January 2018. Of the 140 eligible patients, 82 were randomized to ORIF or functional bracing. Of the 58 patients declining randomization, 42 consented to participate in a nonrandomized cohort in which the patients were able to choose the treatment method. The primary outcome of this study was the Disabilities of the Arm, Shoulder and Hand (DASH) score. Patients in the randomized cohort and the nonrandomized cohort were analyzed separately in 3 groups: those who had (1) initial surgery, (2) successful functional bracing, and (3) late surgery due to failed functional bracing. We used mixed-model, repeated-measures analysis of variance to compare the treatment effect among the 3 groups.

**Results:**

In the randomized cohort, 38 patients had an initial surgical procedure. Of the 44 patients randomized to functional bracing, 30 (68%) healed successfully and 14 (32%) underwent a late surgical procedure. In the nonrandomized cohort, 9 patients preferred an initial surgical procedure. Of the 33 patients preferring functional bracing, 26 (79%) healed successfully and 7 (21%) underwent late surgery. The DASH scores in the randomized cohort and the nonrandomized cohort were 6.8 (95% confidence interval [CI], 2.3 to 11.4) and 12.3 (95% CI, 0.3 to 24.3) for the initial surgery groups, 6.0 (95% CI, 1.0 to 11.0) and 3.4 (95% CI, 0 to 9.3) for the bracing groups, and 17.5 (95% CI, 10.5 to 24.5) and 20.5 (95% CI, 9.4 to 31.6) for the late surgery groups at 2 years.

**Conclusions:**

The results of the randomized cohort and the nonrandomized cohort were comparable and suggest that patients’ treatment preferences are not associated with the treatment outcomes of these injuries.

**Level of Evidence:**

Therapeutic Level I. See Instructions for Authors for a complete description of levels of evidence.

Randomized clinical trials (RCTs) are considered the foundation of high-quality evidence in clinical research. However, RCTs are typically conducted on carefully selected patients, and some eligible patients may not be willing to undergo randomization. This may introduce unintended selection bias, as the randomized patients may not have the same baseline characteristics as those patients who declined randomization. Additionally, treatment decisions ideally include patients’ treatment preferences (shared decision-making), which may be associated with the treatment outcomes.

While conducting an RCT (the FISH [Finnish Shaft of the Humerus] trial) comparing the effectiveness of surgery (open reduction and internal plate fixation [ORIF]) and nonoperative treatment (functional bracing) in the treatment of closed, displaced, humeral shaft fractures^1,2^, we prospectively collected a follow-up cohort that included eligible patients who declined to participate in the RCT (nonrandomized cohort). In our previously reported RCT results^1,2^, patients who were treated initially with functional bracing but then underwent late surgery due to healing problems had inferior outcomes up to the 2-year follow-up. The aim of our study was to compare the results between the randomized cohort and the nonrandomized cohort and to evaluate if the patients’ initial treatment preferences were associated with the treatment outcomes.

## Materials and Methods

### Trial Design, Participants, and Interventions

The published study protocol of the FISH trial provides a comprehensive overview of the trial’s design, rationale, and methodology^3^. Briefly, the FISH trial was a multicenter, randomized clinical superiority trial performed in 2 university hospitals (Helsinki and Tampere) in Finland. The study was registered at ClinicalTrials.gov (NCT01719887), and the institutional review board of the Helsinki and Uusimaa Hospital District approved the study protocol. The study was conducted in accordance with the Declaration of Helsinki. All patients gave written informed consent. Patients were recruited between November 2012 and January 2018. Consenting adult patients (≥18 years of age) with a displaced, closed, humeral shaft fracture were randomized to surgical care with ORIF or nonsurgical care with functional bracing (randomized cohort). Patients were randomly assigned to the study group by a surgeon from the research group using a block size of 4 through a closed-envelope method, employing distinct computer-generated randomization lists for each of the study centers. Exclusion criteria encompassed any past or present conditions that would affect the function of the injured upper limb, pathological fracture, any other concurrent injuries to the same upper limb, other trauma necessitating a surgical procedure (fracture or internal organ, brachial plexus, or vascular injury), insufficient cooperation (for example, due to substance abuse or dementia), multimorbidity (resulting in high anesthesia risk), or polytrauma. The results of the randomized cohort have been published previously^1,2^.

Eligible patients who were unwilling to undergo randomization were given the option to participate in an observational cohort (nonrandomized cohort). They were provided with information about the risks and potential benefits of both surgery and nonsurgical treatment and were allowed to choose their preferred treatment.

In the surgery group, the procedure was performed by an experienced orthopaedic trauma surgeon or under their supervision. For patients in the bracing group, a plaster technician fitted a functional brace to immobilize the humeral shaft fracture, allowing free motion of the shoulder and elbow joints. A structured rehabilitation program was implemented, which included written instructions and visits to a physical therapist at 3 and 9 weeks after the fracture. Follow-up appointments were conducted at the outpatient clinic of the study centers by orthopaedic surgeons and orthopaedic residents at 6 weeks; 3, 6, and 12 months; and 2 years. Patients experiencing issues with healing received treatment based on the treating surgeon’s preference.

### Objective

The objective of this secondary analysis was to compare the randomized cohort and the nonrandomized cohort with 2-year follow-up and explore whether patients’ treatment preference was associated with the outcomes.

### Outcomes

The primary outcome measured in our study was the Disabilities of the Arm, Shoulder and Hand (DASH) score. The DASH score is a commonly utilized patient-reported outcome measure that assesses physical function and symptoms related to the upper limb. Scores range from 0 to 100, in which 0 indicates no disability and 100 represents extreme disability. The minimal important difference (MID) for the DASH score is 10^4,5^.

Secondary outcomes included pain during activities, measured using a 0-to-10 numerical rating scale (NRS); the Constant-Murley score, a common score for evaluating shoulder function (scores range from 0 to 100, with higher scores indicating better function)^6,7^; the 15D quality-of-life instrument^8^; and the proportion of patients in an acceptable symptom state, assessed through the patient’s global assessment of satisfaction with the injured arm’s overall condition and its impact on daily life. Answers were provided on a 7-point Likert scale, with “Very satisfied” and “Satisfied” indicating a patient acceptable symptom state (PASS), and responses of “Somewhat satisfied,” “Neither satisfied nor dissatisfied,” “Somewhat dissatisfied,” “Dissatisfied,” and “Very dissatisfied” indicating an unacceptable state. The proportions of patients achieving the PASS and achieving adequate clinical recovery (defined as a preinjury DASH score plus 10 points [MID]) were assessed.

Questionnaires were distributed at baseline and during each follow-up visit (at 6 weeks; 3, 6, and 12 months; and 2 years). At baseline, we gathered demographic and clinical information and had patients recall their condition before the fracture with the use of the DASH score and the 15D instrument. During each follow-up visit, we evaluated fracture-healing and potential complications, such as implant failure, malunion, and refracture, through both clinical assessment and anteroposterior and lateral radiographs. The range of motion of the shoulder and elbow was measured by a trained physical therapist using a goniometer, and shoulder strength (a component of the Constant-Murley score) was assessed using a calibrated spring balance.

Data on complications, adverse events, and reoperations were gathered from medical reports. Adverse events were categorized as either serious or minor. To determine cases of fracture nonunion and malunion, a committee of 5 experienced orthopaedic surgeons reviewed all radiographs and patient reports. Nonunion was defined as the absence of fracture-bridging callus in 3 of 4 cortices on anteroposterior and lateral radiographs made at least 3 months after the fracture, coupled with clinically confirmed movement at the fracture site.

During the follow-up visits, outcome assessors conducting objective measurements were kept unaware of the treatment group. This was achieved by instructing patients to remove the brace, wear a long-sleeved shirt, and refrain from verbally disclosing their study group.

### Statistical Analysis

This secondary analysis of both the randomized and nonrandomized cohorts from the FISH trial aimed to investigate potential differences in functional outcomes at the 2-year follow-up between patients who chose their treatment method and those who were randomized. The participants were divided into 3 groups for analysis: (1) an initial surgery group, (2) a bracing group that experienced successful healing, and (3) a late surgery group. The last group included patients who initially received functional bracing, but underwent a late surgical procedure to facilitate fracture-healing during the 2-year follow-up period.

To accommodate missing values in the data sets, we conducted comparisons using mixed-model, repeated-measures analysis of variance (ANOVA), assuming missingness at random. Study group, time of assessment, and study site were considered fixed factors, and patients were considered random factors. The model accounted for interactions between the study group and assessment times. Changes from baseline were calculated using the baseline value as a covariate. The model assessed the treatment effect by comparing the DASH score (mean and 95% confidence interval [CI]) among the 3 groups. A similar approach was taken to evaluate secondary outcomes, where applicable (such as the NRS for pain at rest and during activities, 15D, and Constant-Murley score). For categorical response variables (including the proportions of patients achieving the PASS, achieving adequate clinical recovery), the Fisher exact test was used because of issues with convergence in logistic regression analysis. The Fisher exact test was also utilized to calculate relative risk ratios; the method of Katz et al.^9^ was used to obtain 95% CIs for the relative risk ratios. For the comparison of baseline characteristics of the randomized and nonrandomized cohorts, we used the independent sample t test (continuous variables) and the Pearson chi-squared test (categorical variables).

After obtaining the results separately for the randomized and nonrandomized cohorts, we analyzed the results to explore whether there were any differences in the outcomes. An independent statistician performed all of the analyses. Two-sided p values were calculated, and p < 0.05 was considered significant. Data analysis was using Stata (version 15.1; StataCorp).

## Results

### Characteristics of the Patients

**Fig. 1:**
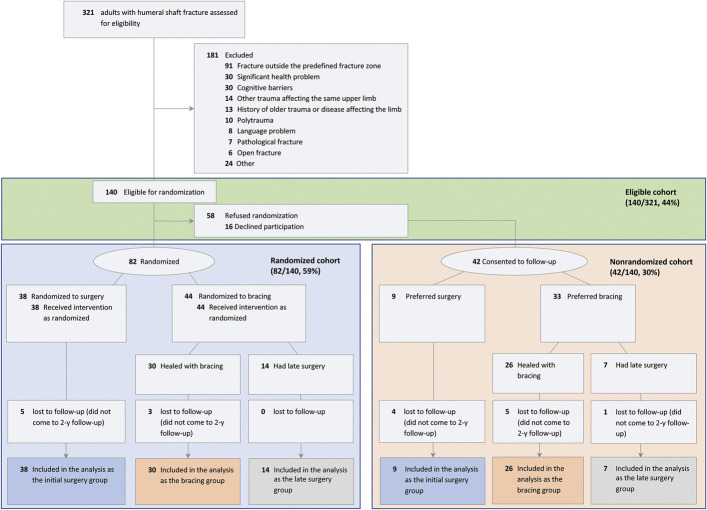
Flowchart of the study. Some patients had >1 reason for being excluded.

Of the 321 patients evaluated for eligibility, 181 were deemed ineligible, a total of 82 patients were randomized, and 58 patients opted out of randomization. Of the patients who opted out, 42 agreed to be part of the nonrandomized cohort. Altogether, 124 (39%) of the 321 assessed patients were included in this analysis (Fig. 1). The 3 groups in the randomized cohort consisted of 38 patients who underwent surgery (initial surgery group), 30 patients who had successful healing using the brace (bracing group), and 14 patients who underwent delayed surgery due to healing problems with bracing (late surgery group). In the nonrandomized cohort, there were 9 patients in the initial surgery group, 26 patients in the bracing group, and 7 patients in the late surgery group. The baseline characteristics of the randomized and nonrandomized cohorts were similar, except that the patients in the nonrandomized cohort more often had a distal shaft fracture (Table I).

**TABLE I:** Baseline Demographic and Clinical Characteristics

Characteristic	Randomized Cohort (N = 82)	Nonrandomized Cohort (N = 42)	P Value*
Age at allocation† *(yr)*	48.9 ± 17.1 (19 to 81)	44.6 ± 17.3 (20 to 83)	0.19
Female sex‡	38 (46.3%)	16 (38.1%)	0.45
Body mass index§ *(kg/m*^*2*^*)*	27.9 ± 5.0	27.3 ± 4.4	0.55
Smoker‡	21 (25.6%)	10 (23.8%)	1.00
Radial nerve palsy‡#	5 (6.1%)	3 (7.1%)	1.00
OTA/AO classification type‡			0.21
A, simple	70 (85.4%)	30 (71.4%)	
B, wedge fragment	11 (13.4%)	11 (26.2%)	
C, segmental	1 (1.2%)	1 (2.4%)	
Fracture location‡**			0.02
Proximal shaft	7 (8.5%)	2 (4.8%)	
Mid-shaft	72 (87.8%)	32 (76.2%)	
Distal shaft	3 (3.7%)	8 (19.0%)	
Injury mechanism‡††			0.77
Low-energy	72 (87.8%)	38 (90.5%)	
High-energy	10 (12.2%)	4 (9.5%)	
Dominant limb injured‡	38 (46.3%)	22 (52.4%)	0.57
Pre-injury scores§			
DASH‡‡	2.6 ± 5.6	3.8 ± 9.4	0.36
15D§§	0.95 ± 0.05	0.94 ± 0.09	0.53

* Two-sided significance with independent-sample t test for continuous variables and 2-sided significance with Pearson chi-square test for variables with proportions. The only significant difference between the groups was in the fracture location.

† The values are given as the mean and standard deviation, with the range in parentheses.

‡ The values are given as the number of patients, with the percentage in parentheses.

§ The values are given as the mean and the standard deviation.

# Patients were categorized as having radial nerve palsy when subtotal or total motor palsy was noted. Normal function, mild motor weakness, or sensory disturbance was categorized as no radial nerve palsy. Patients were stratified according to radial nerve status.

** Fracture location was classified by the third of the diaphysis in which the center of the fracture was located.

†† Injury mechanism was classified as high-energy if the height of a fall was over standing height or if the fracture was sustained in a traffic accident.

‡‡ The DASH score is a widely used and validated tool assessing upper-extremity-related deficits and symptoms in daily life reported by the patient. The instrument consists of 30 items. The range of the score is from 0 (no disability) to 100 (extreme disability). Values of <10 points are comparable with the mean value in a randomly selected population between 20 and 60 years of age. Ten points is generally regarded as a minimal important difference in the DASH score. At baseline, the patient was asked to report the situation just before the fracture.

§§ The 15D instrument is a generic health-related quality-of-life instrument comprising 15 dimensions. The maximum 15D score is 1 (full health), and the minimum score is 0 (death). Values of >0.9 are comparable with a randomly selected Finnish population of individuals ≥30 years of age. At baseline, the patient was asked to report the situation just before the fracture.

Eighteen patients (8 [10%] of the 82 in the randomized cohort and 10 [24%] of the 42 in the nonrandomized cohort) were lost to follow-up at 2 years, 9 from the initial surgery group (5 from the randomized cohort and 4 from the nonrandomized cohort), 8 from the bracing group (3 from the randomized cohort and 5 from the nonrandomized cohort), and 1 from the late surgery group (from the nonrandomized cohort).

### Primary Outcome

**Fig. 2:**
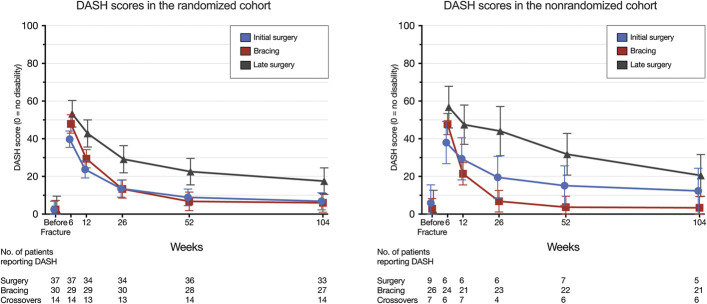
DASH scores in the randomized cohort and the nonrandomized cohort of the FISH trial. In the initial surgery group of the randomized cohort, 1 patient's baseline DASH score was missing, reducing the number of patients from 38 to 37. The error bars indicate the 95% CIs.

The DASH scores in the randomized cohort and the nonrandomized cohort were 6.8 (95% CI, 2.3 to 11.4) and 12.3 (95% CI, 0.3 to 24.3) for the initial surgery groups, 6.0 (95% CI, 1.0 to 11.0) and 3.4 (95% CI, 0 to 9.3) for the bracing groups, and 17.5 (95% CI, 10.5 to 24.5) and 20.5 (95% CI, 9.4 to 31.6) for the late surgery groups at 2 years. The results at the other time points are presented in Figure 2 and Table II.

**TABLE II:** DASH Scores at Different Time Points*

DASH Scores	Initial Surgery Group	Bracing Group	Late Surgery Group	Between-Group Difference		
				Initial Surgery vs. Bracing	Initial Surgery vs. Late Surgery	Bracing vs. Late Surgery
Randomized cohort (n = 82)	n = 38	n = 30	n = 14			
6 weeks	39.8 (35.4 to 44.1)	47.9 (43.0 to 52.7)	53.3 (46.2 to 60.3)	−8.1 (−14.7 to −1.5)	−13.5 (−21.8 to −5.3)	−5.4 (−14.0 to 3.2)
12 weeks	23.7 (19.2 to 28.2)	29.4 (24.5 to 34.3)	42.8 (35.6 to 50.0)	−5.7 (−12.3 to 1.0)	−19.1 (−27.6 to −10.6)	−13.4 (−22.1 to −4.7)
6 months	13.5 (9.0 to 18.0)	13.3 (8.5 to 18.2)	29.1 (21.9 to 36.3)	0.2 (−6.4 to 6.8)	−15.6 (−24.1 to −7.1)	−15.8 (−24.5 to −7.1)
12 months	8.9 (4.5 to 13.3)	6.7 (1.8 to 11.7)	22.6 (15.5 to 29.6)	2.1 (−4.5 to 8.8)	−13.7 (−22.0 to −5.4)	−15.8 (−24.4 to −7.2)
2 years	6.8 (2.3 to 11.4)	6.0 (1.0 to 11.0)	17.5 (10.5 to 24.5)	0.8 (−6.0 to 7.6)	−10.7 (−19.1 to −2.3)	−11.5 (−20.1 to −2.9)
Nonrandomized cohort (n = 42)	n = 9	n = 26	n = 7			
6 weeks	38.0 (26.8 to 49.2)	47.8 (42.2 to 53.4)	56.8 (45.7 to 67.9)	−9.8 (−22.4 to 2.9)	−18.8 (−34.7 to −3.0)	−9.0 (−21.4 to 3.4)
12 weeks	29.4 (18.2 to 40.6)	21.4 (15.5 to 27.4)	47.5 (37.1 to 57.9)	8.0 (−4.8 to 20.7)	−18.1 (−33.5 to −2.7)	−26.1 (−38.0 to −14.1)
6 months	19.5 (8.3 to 30.7)	6.8 (1.1 to 12.6)	44.1 (31.0 to 57.1)	12.7 (0.0 to 25.4)	−24.6 (−41.9 to −7.3)	−37.2 (−51.5 to −23.0)
12 months	15.1 (4.6 to 25.7)	3.7 (0 to 9.5)	31.8 (20.7 to 42.8)	11.4 (−0.7 to 24.0)	−16.7 (−32.0 to −1.3)	−28.1 (−40.6 to −15.6)
2 years	12.3 (0.3 to 24.3)	3.4 (0 to 9.3)	20.5 (9.4 to 31.6)	9.0 (−4.6 to 22.5)	−8.2 (−24.6 to 8.2)	−17.2 (−29.7 to −4.6)

* The point estimates are derived from the mixed-model, repeated-measures ANOVA model using all available data. Patients who were able to follow the protocol until healing are included in the surgery and bracing groups according to their randomization. The patients in the late surgery group were randomized to bracing but underwent secondary surgery to promote the healing of the fracture during the follow-up. The DASH score is a widely used and validated tool assessing upper-extremity-related deficits and symptoms in daily life reported by the patient. The instrument consists of 30 items. The range of the score is from 0 (no disability) to 100 (extreme disability). Values below 10 points represent the mean found in a randomly selected general population 20 to 60 years of age. Ten points is generally regarded as a minimal important difference in the DASH score. The values are given as the mean, with the 95% CI in parentheses.

Significant and clinically meaningful differences favoring the initial surgery group were observed between the initial surgery and late surgery groups in both the randomized and nonrandomized cohorts at all of the follow-up points, with the exception of the nonrandomized cohort at 2 years (initial surgery minus late surgery, −8.2 points [95% CI, −24.6 to 8.2 points]) (Table II). The differences between the successful bracing and late surgery groups were significant and clinically important, in favor of the successful bracing group, both in the randomized cohort (bracing minus late surgery, −11.5 points [95% CI, −20.1 to −2.9 points]) and in the nonrandomized cohort (−17.2 points [95% CI, −29.7 to −4.6 points]) at 2 years.

### Secondary Outcomes

**Fig. 3:**
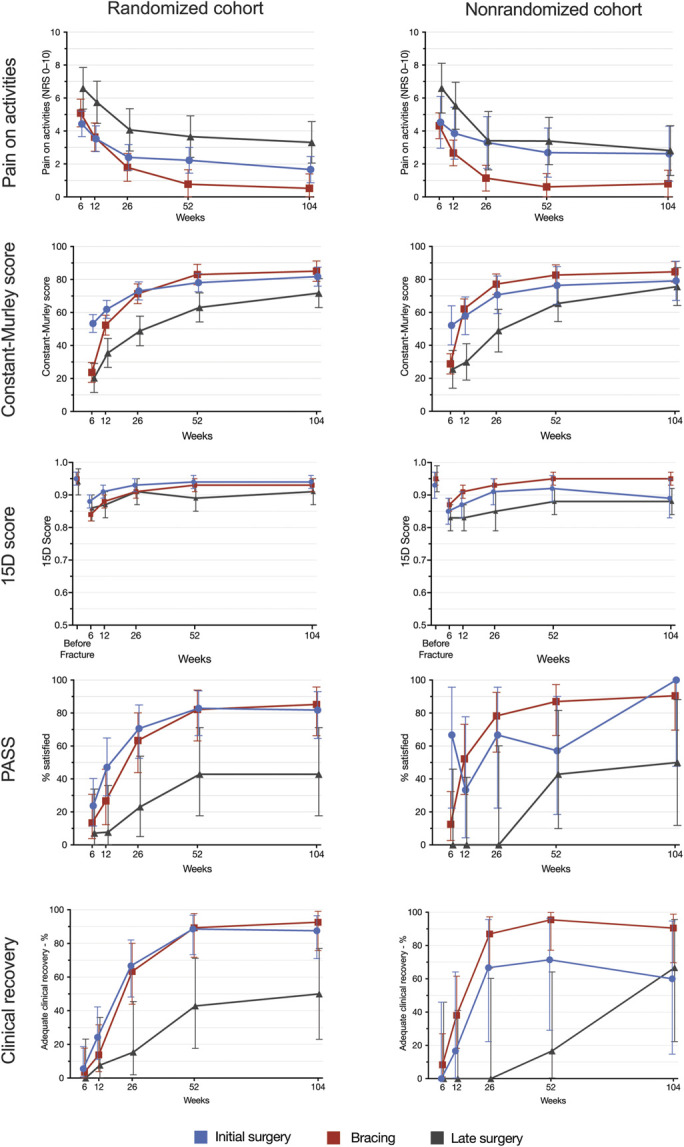
Secondary outcomes of the randomized cohort and the nonrandomized cohort. PASS = patient acceptable symptom state. The error bars indicate the 95% CIs.

Figure 3 summarizes the secondary outcomes. At the 2-year mark, we observed significant differences between the late surgery group and the other treatment groups in most secondary outcomes, favoring the initial surgery and bracing groups in both the randomized and nonrandomized cohorts.

### Adverse Events

Adverse events and reasons for late surgery are listed in Table III. None of the patients in the initial surgery group of the randomized cohort had to undergo late surgery, compared with 3 patients in the initial surgery group of the nonrandomized cohort (p = 0.005).

**TABLE III:** Adverse Events and Crossovers

Description	Initial Surgery Group		Bracing Group		Late Surgery Group	
	Randomized Cohort (N = 38)	Nonrandomized Cohort (N = 9)	Randomized Cohort (N = 30)	Nonrandomized Cohort (N = 26)	Randomized Cohort (N = 14)	Nonrandomized Cohort (N = 7)
Serious adverse event						
Cardiovascular event	1		1			
Minor adverse event						
Fracture nonunion		2	2		9	6
Refracture		1			1	
Secondary temporary radial nerve palsy	4	1			1	
Superficial wound infection	2				1	
Wound seroma	1					
Shoulder adhesive capsulitis	1		1			
Loss of reduction					1	
Sensory disturbance in the forearm					1	1
Reason for late surgery						
Nonunion		2			9	6
Loss of reduction		1			1	
Refracture					1	
Intolerable pain at the fracture site					1	
Not tolerating the bracing					2	
Uncertain continuity of radial nerve in electroneuromyography						1

## Discussion

In this secondary analysis of the FISH trial, we compared the data of the randomized patients with those of the eligible patients who declined randomization (nonrandomized cohort) to evaluate if patients’ treatment preferences were associated with treatment outcomes. As in our previously reported RCT results^2^, patients in the randomized cohort who were treated initially with functional bracing but underwent a late surgical procedure because of healing problems had inferior outcomes, and the same trend was observed in the nonrandomized cohort. This suggests that the inferior results at the 2-year follow-up of the late surgery group were not related to patients’ treatment preferences.

The RCT-in-cohort design allowed us to address a possible limitation of our RCT and RCTs in general, namely, the existence of recruitment bias. Recruitment bias arises from the fact that often only a proportion of all eligible patients are randomized, as some of the patients do not accept randomization because they favor one treatment modality over the other. This affects the generalizability of the results, as one can only guess to what extent the participants of the RCT are representative of the target population studied. We found 140 of 321 adult patients with humeral shaft fractures to be eligible for randomization, yielding an eligibility rate of 44%. Of the 140 eligible patients, we included 124 (an 89% inclusion rate) in this study: 82 randomized (a 66% randomization rate) and 42 nonrandomized participants. We suggest that the terms “eligibility rate” (the proportion of eligible patients among all assessed patients), “inclusion rate” (the proportion of patients willing to participate in the study among all eligible patients), and “randomization rate” (the proportion of patients consenting to randomization among all included patients) could be valuable tools to evaluate the generalizability of trial results. In this study, both the randomized and nonrandomized cohorts showed comparable baseline characteristics (Table I). In addition, the treatment protocols and treating physicians of the randomized cohort and the nonrandomized cohort were the same. This highlights the generalizability of our results and ensures the comparability of the cohorts in this secondary analysis.

To our knowledge, no previous study has shown a similar comparison on patients with humeral shaft fractures. Several RCTs (on appendicitis^10^, Achilles tendon rupture^11^, ankle fracture^12^, diverticulitis^13^, and spine surgery^14^) have included concurrent observational cohorts to assess the generalizability of the findings by comparing the RCT results with those of the observational cohort results. As in our study, the baseline characteristics of the randomized and the nonrandomized cohorts were comparable in all but the diverticulitis study. In line with our study, most patients declining randomization chose nonoperative treatment in the Achilles tendon rupture and ankle fracture studies. In the appendicitis study, approximately one-half of the patients declining randomization chose nonoperative treatment, and most of the patients in the spine surgery study chose surgical treatment. In accordance with our results, there was no significant difference between the randomized and nonrandomized cohorts in the appendicitis, ankle fracture, and spine surgery studies.

Although our study provides valuable insights into the outcomes of patients with closed, isolated fractures within the context of our RCT, it is essential to acknowledge certain limitations. First, the numbers of participants in the nonrandomized cohort, particularly within the plating group, were relatively small, raising potential concerns about the robustness of the conclusions drawn from the analysis. Second, we could not make any statements regarding compliance with nonsurgical treatment, as we lacked information on the reliability of brace usage. However, because of the pragmatic nature of our study, the outcomes should be considered representative of real-world scenarios. Additionally, the post hoc nature of the comparison between the randomized and nonrandomized cohorts suggests that our findings should be considered exploratory. Also, our findings only apply to patients eligible for our RCT; the exclusion of patients with more complex injuries limits the generalizability of our results. Notably, the loss to follow-up was higher in the nonrandomized cohort, reaching 24%, potentially introducing selection bias. Therefore, although our study contributed valuable insights, the limitations highlight the importance of further research with larger sample sizes and diverse patient populations to enhance the robustness and generalizability of our findings.

In conclusion, our study compared the randomized and nonrandomized cohorts of the FISH trial, assessing the effectiveness of surgery and functional bracing in treating humeral shaft fractures in adults. The results of the nonrandomized cohort were in line with our previously published RCT and suggest that patients’ treatment preferences are not associated with the treatment outcomes for these injuries.

## Data Availability

A **data-sharing statement** is provided with the online version of the article (http://links.lww.com/JBJSOA/A707).
